# The Treatment of Inguinal Hernia in Children

**Published:** 1894-07

**Authors:** Samuel E. Milliken

**Affiliations:** 36 West 59th Street; New York


### THE TREATMENT OF INGUINAL HERNIA IN CHILDREN.

By SAMUEL E. MILLIKEN, M.D., New York.

So much has been written on the "radical cure" of hernia during the past few years, that surgeons seem to have neglected the mechanical treatment, which often effects a cure, if persistently and intelligently carried out during childhood. Before recommending the radical operation, the following points should be considered, viz:

1. The age of the patient.
2. The care received at home.
3. The length of time a truss has been worn.
4. Complications which prevent the satisfactory mechanical treatment.
5. The general condition of the patient.

*First:* While it is true that the radical operation promises better results, as to ultimate cure during childhood, any such procedures should not be attempted before the fourth year of age, unless some special indications for the same be present, until the treatment by truss has been attempted.

*Second:* Neglect at home often makes conservative measures useless; this is particularly true of the lower walks of life. For this reason, some form of apparatus should be employed which does not necessitate removal during the bath.

*Third:* Before the child begins to walk, if it be strong and healthy, it is possible to effect a cure in a few weeks, while, after the first year, it should be worn for at least six months before an attempt be made to leave off the truss, and not infrequently it requires a year to effect a permanent cure.

*Fourth:* Adherent omentum, or a retained testis, are two conditions which indicate the radical operation. Should the child be restless and misplace the truss, it will often be found that the hernia protrudes, and thus delays or prevents the accomplishment of the desired result. Of course a hernia that cannot be retained, and becomes incarcerated, should be operated upon without delay, for fear of strangulation taking place.

*Fifth:* Any organic disease, such as a heart lesion, may contra-indicate the use of an anesthetic, and thus make the operation more grave.

*Methods Employed:* The injection of white oak bark into the canal can only be considered an adjuvant to the truss treatment, as it rarely effects a cure alone. The "open method," where the wound is allowed to granulate, although only advocated a few years ago, has become almost obsolete, as the new tissue tends toward self-destruction, and often leaves the patient with a worse hernia than at first existed.

Of the many other methods advocated, such as removing the sac and suturing the pillars of the ring, the percentage of cures is small. The reconstruction of the inguinal canal, as suggested and performed by Bassini, offer the best results, but it is essential that primary union be obtained. "Bassini's method" consists, (*a*) in slitting up the inguinal canal to well above the internal ring, then lifting the cord structures out of their bed; (*b*) the isolation of the sac from the cord, and the highest possible ligation; (*c*) the re-building of the posterior wall of the canal by bringing together the conjoined tendon on the upper, and the shelving process of Poupart's ligament on the lower side, with interrupted, kangaroo-tendon sutures; (*d*) re-establishing the anterior wall of the canal, with a continuous suture of the kangaroo-tendon; and (*e*) closing the skin wound with catgut.

The patient should remain in bed for two weeks, and, if strict asepsis has been observed, the wound should heal under one dressing. No drainage is employed.

36 *West 59th Street.*

---

A CHINESE PRESCRIPTION FOR WRY-NECK.—Obtain a rooster exactly six months old, place him in a cage facing north, feed him with wheat ripened during the full moon, collect his excrement passed on the sixth day, mix it with sherry and take a wineglassful of the resulting mixture three times a day.—*Medical Press.*

